# The Correlation between Phospholipase C Epsilon (PLCE1) Gene Polymorphisms and Risk of Gastric Adenocarcinoma in Iranian Population 

**Published:** 2019-07-01

**Authors:** Ramin Shekarriz, Sahar Faghani, Alireza Tafazoli, Mohammad Bagher Hashemi-Soteh

**Affiliations:** 1 Department of Hematology and Oncology, Gastrointestinal Cancer Research Center, Mazandaran University of Medical Sciences, Sari, Iran; 2 Department of Internal Medicine, Mazandaran University of Medical Sciences, Sari, Iran; 3 Department of Biochemistry, Biophysics and Genetics, Faculty of Medicine, Mazandaran University of Medical Sciences, sari, Iran; 4 Immunogenetic Research Center, Cell and Molecular Research Center, Faculty of Medicine, Mazandaran University of Medical Sciences, Sari, Iran

**Keywords:** Gastric cancer, Iranian population, *Phospholipase C Epsilon (PLCE1)* gene, Single nucleotide polymorphisms

## Abstract

**Background:** *Phospholipase C epsilon 1* (*PLCE1*) gene harbors different single nucleotide polymorphisms (SNPs), which can be correlated with the risk of different types of cancers. In this case-control study, the relationship between rs2274223 (A>G), a single nucleotide polymorphism in *phospholipase C epsilon *gene, (*PLCE1*) and gastric cancer was evaluated among Iranian patients.

**Materials and Methods:** The *PLCE1* rs2274223 polymorphism was genotyped in 60 patients with gastric cancer and 69 control subjects using polymerase chain reaction and restriction fragment length polymorphisms (PCR-RFLP) methods. Clinical and pathologic parameters such as tumor characteristics and disease stage were also recorded.

**Results:** There were 48 (80%) male patients and 45 (65.5%) healthy male individuals (p=0.077). About 34 (56.6%) patients were smokers. A family history of gastric cancer was found in 21 (35%) cases. GG genotype was observed among 15% of patients and 8.7% of normals, respectively. There was no significant difference between the AA and AG genotypes. Also, there were no significant correlations between AA, AG or GG genotypes and the risk of gastric cancer, gender, tumor size, tumor stage, grade, as well as tumor location and metastasis.

**Conclusion: **The *PLCE1* rs2274223 polymorphism was not correlated with gastric cancer in Iranian population. However, a further comprehensive study with larger sample sizes is needed.

## Introduction

 Gastric cancer is now considered as one of the most common cancers and leading cause of cancer death (more than 700,000 deaths each year) throughout the world^1^^,^^2^. Therefore, there are still urgent needs for early diagnosis and prevention of it. Adenocarcinoma gastric cancer is a multifactorial disease and various factors may play role in it, including positive family history, *Helicobacter pylori* infection, nutrition, smoking, and alcoholic consumption^        3 ^.

Genetic background is considered as one of the significant causes of gastric cancer^        4 ^. Recent evidence has shown that genetic variants, especially single nucleotide polymorphisms (SNPs), can mediate the effect of environmental risk factors through modifying functions of various signaling pathways involved in gastric carcinogenesis^5^. Genome Wide Association Studies (GWAS) provided good opportunity to identify potential candidates for single nucleotide polymorphisms. Recent GWAS have revealed that *Phospholipase C epsilon 1* gene, *PLCE1*, which is located on chromosome 10q23, participates in cell growth, differentiation and gene expression, as well as induces small GTPases such as Ras, Rap and Rho families. Different SNPs in *PLCE1 *gene are shown to have correlation with the risk of different type of cancers including esophageal and gastric ^6^^-^^8^ . Rs2274223 (A>G), located in exon 26 of the *PLCE1* gene, can cause the amino acid change, histidine (His) to arginine (Arg) at the codon position of 1927 of PLCE1protein. The *PLCE1* gene encodes a phospholipase C that hydrolyses of Polyphatidylinositol to inositol 1,4,5-trisphosphate and 4,5-diacylglycerol^   8 ^. This phospholipase also regulates a variety of proteins such as the protein kinase C (PKC) isoenzymes and the proto-oncogene Ras^   9 ^. Recent studies have identified a new susceptibility for single nucleotide polymorphism (SNP) (rs2274223: A5780G), located in exon 26 of *PLCE1*, which may correlate with the risk of esophageal and gastric cancers^10^^,^^11^  .For instance, Wang et al. reported a relatively high correlation between esophageal squamous cell carcinoma and rs2274223 A>G polymorphism in Chinese population ^        12 ^. In another study, Abnet et al. demonstrated a close relationship between rs2274223 polymorphism and increased risk of gastric adenocarcinoma and esophageal squamous cell carcinoma in Chinese population^        13 ^. However, inconsistent results are also reported and several studies did not report any correlation between this polymorphism and gastric cancer^10^^, ^^11^.

In this study, we attempted to evaluate the correlation between the *PLCE1* (rs2274223 A>G) polymorphism and the risk of gastric adenocarcinoma in northern Iran. Furthermore, it should be mentioned that this is the first study to date to assay the correlation between this polymorphism and gastric adenocarcinoma in Iran.

## MATERIALS AND METHODS

**Subjects**

60 patients with gastric adenocarcinoma and 69 healthy individuals who referred to Imam Hospital at the Mazandaran University of Medical Sciences (Sari-Iran) between 2016 and 2017 were recruited in this study. The Sample size was similar to the previous studies^        14 ^. This case-control study was approved by the Research Board Committee and Ethics Committee at the Mazandaran University of Medical Sciences. Written consent form was signed by all individuals. A questionnaire form, containing demographic data was also filled by all patients before laboratory examinations. Clinical and pathological findings of the patients, including tumor size, disease stage, tumor grading, metastasis status, and lymph node involvement were also evaluated. Tumors were graded according to the WHO Classification of Tumors of the Digestive System^15^. Healthy controls were those who were referred to our hospital for check-up and showed no abnormalities in physical examination or laboratory tests. These individuals also did not have any considerable history of cancer or related diseases. The control subjects were matched to the patients based on their age and sex. 

Inclusion criteria included: (i) the presence of confirmed gastric adenocarcinoma in patients; (ii) availability of complete clinical and pathological information related to the patients. Meanwhile, exclusion criteria included: (i) the presence of tumor in other places such as esophagus; (ii) previous treatments such as chemotherapy or radiotherapy (iii) history of other chronic diseases such as diabetes mellitus, liver disease, etc.

**DNA extraction**

Five to ten ml blood samples were collected from the antecubitalvein of patients and healthy donors using EDTA containing tube. Genomic DNA was extracted from 3 ml of blood sample using the salting out method^   16 ^. Quantity and quality of extracted DNAs were determined using a NanoDropND-2000 spectrophotometer (Thermo Sci., Newington, NH). Isolated DNAs were stored at 20^o^ C until further analysis. 

**SNP Selection**

The functional rs2274223 SNP was selected in *PLCE1 *gene for genotyping based on previous published data and the NCBI dbSNP database (http://www.ncbi.nlm.nih.gov/build 131).

**PCR primers and amplification conditions**

Exon 26 of PLCE1 gene was amplified using polymerase chain reaction followed by restriction fragment length polymorphisms (PCR-RFLP) methods to identify genotypes of rs2274223 A>G polymorphism. An appropriate pair of primers, including forward (F-5ʹ CCTACAATCACTTACTTTTTAAAC3ʹ and reverse primers (R-5ʹ ATACAAGATCTTCGAAGTGA3ʹ) were applied (Macrogene, South chorea). A 379-bp fragment was achieved by PCR amplification in a total volume of 25 μl consisted of approximately 20 ng of genomic DNA (1-2μl), 2 μM of each primer (0.7 μl), 0.1 mM each dNTP (0.5 μl), 1× PCR buffer (2.5 μl), 1.5 mM MgCl_2 _(0.7 μl), 0.5 unit of Taq polymerase (0.2 μl) and DDW (19.3 μl) (Cinnagen, Iran). The DNA thermal cycler device was programmed with the following amplification conditions: initial denaturation step of 95°C for 5 min;35 cycles of 95°C for 40s (denaturation), 58.5°C for 40s (annealing), and 72°C for 40s (extension), and final extension step of 72°C for 10 min. Amplified PCR product was analyzed by 2% gel agarose gels (Kawsar Biotechnology Company, Iran) stained with ethidium bromide (Merck, Germany), visualized with UV illumination.

**Genotyping using PCR-RFLP**

**Figure 1:**
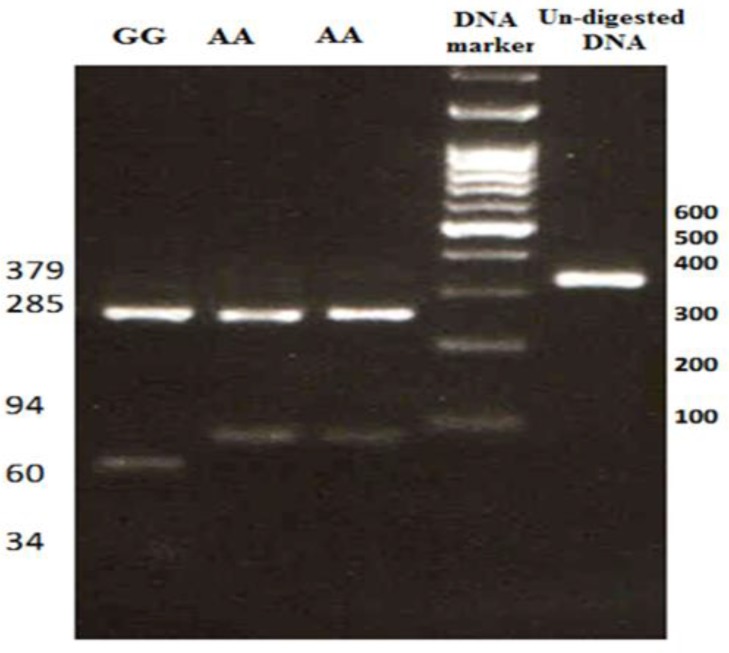
Genotyping of rs2274223 through digestion of PCR product with *AciI* restriction enzyme. The AG genotype is not showed in this figure. The 34-bp fragment cannot be observed here because of small size

The 397 bp PCR product was digested using *AciI*restriction enzyme (0.3µl) (Thermo Fisher Scientific, US) overnight at 37° C. The digested products were then separated on 2% agarose gel electrophoresis, which was stained by safe stain (Sinaclone, Iran) and visualized with UV illumination. The A allele has a restriction site and produces two bands, 285 bp and 94 bp, while replacement of A nucleotide with G creates a new *AciI* restriction site in 94-bp fragment and produces a 60-bp and a 34-bp fragments. Therefore, AA genotype produces 94-bp and 285-bp; GG genotype produces 34-bp, 60-bp and 285-bp and AG genotype produces 34-bp, 60-bp, 94-bp, and 285-bp after enzyme digestion, respectively (Figure 1).

**Statistical analysis**

Demographic and clinical characteristics of all patients were reported as means±SD. An independent student t-test was also considered to compare the mean of age between the patients and control groups. Descriptive statistics as well as Pearson’s Chi-Square test were used to compare the frequency of each genotype between different groups. Data were analyzed using SPSS software (version 19) and a p<0.05 was considered as significant. 

## Results

 The basic demographic characteristics of all patients are summarized in Table 1. In total, 60 patients (48 males and 12 females) and 69 healthy individuals (45 males and 24 females) were entered into the study. The mean of age in the patient and control group was 64.4±11.5 and 56.9±9.5 years, respectively. More than half of the patients (56.66%) were active smokers and 35% of patients had a family history of gastric cancer (Table 1).

**Table 1:** Clinical and basic demographic characteristics of patients

**Variables**		**Patients (n=60)**
Age	64.4±11.5
Gender	
Male (n)	48 (80%)
Female (n)	12 (20%)
Smoker	
Yes	34 (56.66%)
No	26 (43.33%)
Family history	
Yes	21 (35%)
No	39 (65%)
^*^Clinical features	
Bleeding	8 (6.2%)
Obstruction	9 (7%)
Abdominal pain	31 (24%)
Dyspepsia	24 (18.6%)
Anemia	4 (3.1%)

There are overlaps for some data. Eight patients had abdominal pain and dyspepsia, 2 patients had bleeding and anemia, 3 patients had dyspepsia and bleeding, and 3 patients had abdominal pain and obstruction.

Tumor characteristics and disease stage of all patients are presented in Table 2. Ulcerative pattern was the most common tumor type among the patients (58.3%). About 51.7% of tumors were classified as poor differentiated and 48.3% as moderately differentiated. Lymph node invasion was found among the majority of the patients (63.3%). About 61.7% of cases had tumor metastasis. The percentage of patients with stages I, II, III and IV were 6.7%, 35%, 20% and 38.3%, respectively (Table 2).

**Table 2:** Tumor characteristics in patients

**Variables **	**Results **
^*^Tumor location	
Cardia	10 (16.7%)
Fundus	24 (40%)
Body	40 (66.3%)
Antrum	24 (40%)
^*^Tumor type	
Polypoid	14 (23.3%)
Ulcerative	35 (58.3%)
Diffuse (infiltrative)	26 (43.3%)
Tumor grade	
Well-differentiated	-
Moderately differentiated	29 (48.3%)
Poorly differentiated	31 (51.7%)
Lymph node invasion	
Yes	38 (63.3%)
No	22 (36.7%)
Perineural invasion	
Yes	25 (41.7%)
No	35 (58.3%)
Tumor invasion	
1	1 (1.7%)
2	26 (43.3%)
3	28 (46.7%)
4	5 (8.3%)
Lymph node involvement	
0	6 (10%)
1	17 (28.3%)
2	28 (46.7%)
3	9 (15%)
Metastasis	
Yes	37 (61.7%)
No	23 (38.3%)
Stage	
I	4 (6.7%)
II	21 (35%)
III	12 (20%)
IV	23 (38.3%)

There are overlaps for some data. For tumor location: one patient had tumor in cardia and antrum, 2 patients had tumor in cardia and body, 10 patients had tumor infundus and body, 7 patients had tumor in body and antrum, 18 patients had tumor in antrum, body and fundus. For tumor type: 7 patients had ulcer and diffuse, 3 patients had polypoid and ulcer, 3 patient had polypoid and diffuse, and 2 patients had ulcer, diffuse and polypoid. 

The frequency of AA, AG and GG genotypes among the control and patient groups is shown in Table 3. There was no significant difference in the frequency of these genotypes between the patient and control group (p=0.36). While AA was the most common genotype in the patients (60%) and controls (71%), GG genotype was the least common in both groups (Table 3).

**Table 3:** Comparison of genotype and allele frequency between the control and gastric cancer patient groups (n=129)

**Genotypes **	**Control ** **(n=69)**	**Patients ** **(n=60)**	**P **	**OR**	**95% CI**	**P **
AA	49 (71%)	36 (60%)	0.36			
AG	14 (20.3%)	15 (25%)		1.458	0.626- 3.398	0.382
GG	6 (8.7%)	9 (15%)		2.042	0.667- 6.251	0.211
Allele						
A	0.81 (81%)	0.73 (73%)	0.81			
G	0.19 (19%)	0.27 (27%)				

As indicated in Table 3, the variant rs2274223 was no significantly associated with gastric cancer [OR=2.04; CI 0.667-6.251; p=0.221 (for the GG genotypes *vs.* the AA genotypes) and OR=1.45; CI 0.626-3.398; p=0.382 (for the GA genotypes *vs.* the AA genotypes)]. 

**Figure 2:**
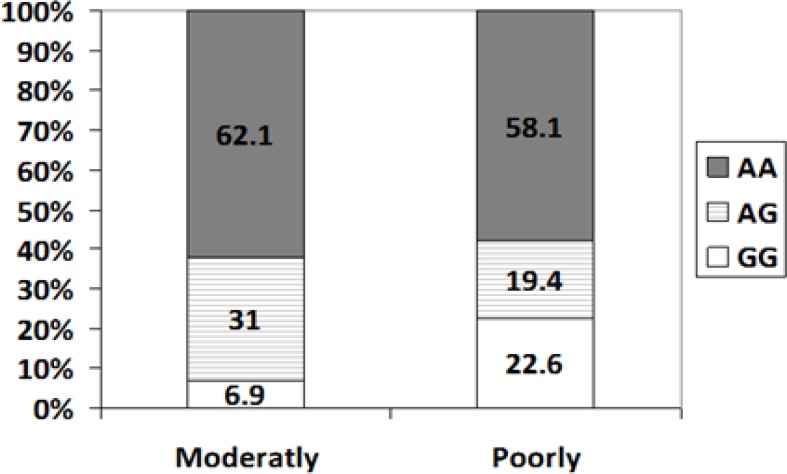
Relationship between the frequency of GG, AG as well as AA genotypes and tumor grade

The Correlation between the frequency of GG, AG as well as AA genotypes and tumor grading is depicted in Figure 2. There was no significant difference in the frequency of each genotype between patients with poorly or moderately differentiated tumor grading. Nevertheless, the frequency of AG genotype among moderately differentiated group (31%) was approximately greater than that in poorly differentiated group (19.4%). Additionally, the frequency of GG genotype in poorly differentiated group (22.6%) was partly greater than moderately differentiated group (6.9%). Also, allele frequency was calculated using genotype frequencies results. Allele A was 73% among patients and 81% among controls, respectively. Allele G was 27% among patients and 19% among controls, respectively (Table 3). There was no significant difference in the allele frequency between the patient and control group (p=0.81). According to the data presented in Table 3, there was deviation from Hardy-weinberg equilibrium for genotypes in controls (P <0.017). This deviation from the HW equilibrium was probably due to the small sample size. 

**Figure 3:**
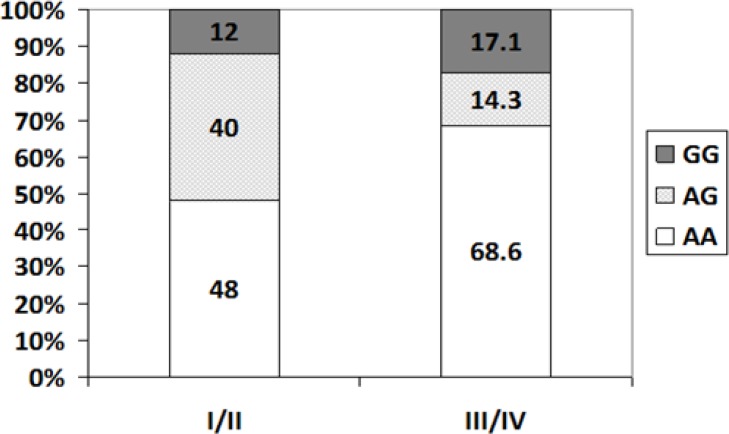
Comparison of the frequency of GG, AG and AA genotypes between patients with I/II and III/IV disease stages

The relationship between the frequency of GG, AG and AA genotypes and disease stages is depicted in Figure 3 and Table 4. There was no significant difference in the frequency of GG, AG and GG genotypes among different stages (p=0.089). However, the AA genotype was the most frequent genotype in patients with disease stage II (18.33%), III (13.33%), and IV (26.66%) compared to the other genotypes (Figure 3 and Table 4). 

**Table4:** Correlation between the frequency of GG, AG and AA genotypes and disease stages

		**Polymorphism**			**P **
		**AA**	**AG**	**GG**	
Stage	I	1 (1.66%)	2 (3.33%)	1 (1.66%)	0.089
	II	11 (18.33%)	8 (13.33%)	2 (3.33%)	
	III	8 (13.33%)	0	4 (6.66%)	
	IV	16 (26.66%)	5 (8.33%)	2 (3.33%)	

The correlation between lymph node involvement, metastasis and tumor location among all three different genotypes is presented in Table 5. We could not find a significant difference in the percentage of lymph node involvement (p=0.77), metastasis (p=0.42) and tumor place (p=0.77) among individuals with GG, AG, and AA genotypes. 

**Figure 4:**
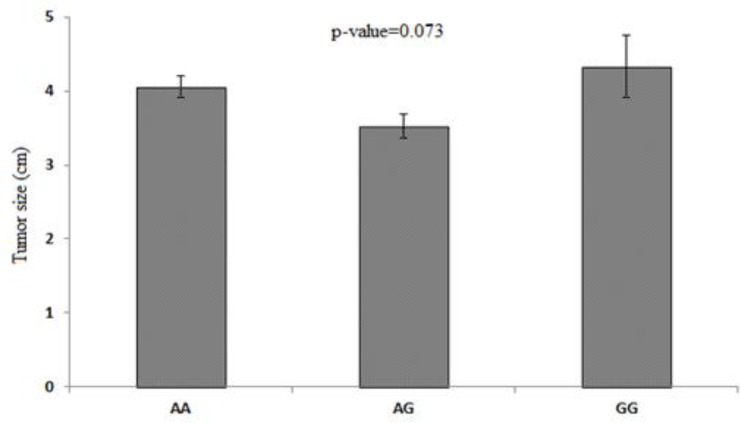
Correlation between tumor size and different genotypes

The mean of tumor size among patients with three different genotypes is presented in Figure 4. We could not find a significant difference between the tumor size and these three genotypes (p=0.073). Nevertheless, the mean of tumor size in GG genotype (4.33±1.25) was greater than that in the AA (4.06±0.89) and AG (3.53±0.61) groups.

**Table 5:** Lymph node involvement, metastasis and tumor place in three different genotypes

**Tumor status**	**GG**	**AG**	**AA**	**P**
Lymph node involvement				
No	1 (16.7%)	2 (33.3%)	3 (50%)	0.77
Yes	33 (61.1%)	13 (24.1%)	8 (14.8%)	
Metastasis				
No	7 (18.9%)	10 (27%)	20 (54.1%)	0.42
Yes	2 (8.7%)	5 (21.7%)	16 (69.6%)	
Tumor place				
Cardia	1 (10%)	2 (20%)	7 (70%)	0.77
Non- Cardia	29 (58%)	13 (26%)	8 (16%)	

There was no significant correlation between tumor size and all three different genotypes (p=0.073)

## Discussion

 In this case-control study, we considered the correlation between a potentially functional SNP of *PLCE1 *(rs2274223) and the risk of gastric adenocarcinoma in Iranian population. Although a large number of studies found a significant relationship between rs2274223 A>G and disease stage and tumor characteristics in different types of cancers, our findings revealed that there is no relationship between different genotypes of AA, AG and GG with disease stage. We also could not found a significant correlation between these genotypes with age, gender, tumor characteristics such as tumor size, metastasis, grading, tumor location, and other factors. 

Numerous studies have investigated several potentially functional SNPs of *PLCE1* in various cancers such as gastric, esophagus and colorectal cancers^   17 ^. For example, Abnetet al.^        13 ^ investigated the correlation between the rs2274223 A>G and the risk of esophagus cancer in 2,240 patients with gastric cancer. They found a close relationship between this SNP and the risk of esophagus cancer (OR=1.31). In another study, Wang et al. considered the correlation between the *PLCE1 *rs2274223 and the risk of gastric cancer in 1059 patients with pathologically confirmed gastric adenocarcinoma and 1240 frequency-matched healthy controls. They observed a significant correlation between this SNP and the risk of gastric cancer (OR = 1.26)^        5 ^. In another similar study, Wu et al.^        18 ^ investigated the relationship between the rs2274223 with esophagus cancer and its tumorigenesis in 2,031 Chinese patients with gastric cancer and 2,044 controls. They identified a significant correlation between the SNP and esophagus cancer and its tumorigenesis. Ezgi*et al*. also reported a significant correlation between rs2274223 and the risk of colorectal cancer in Turkish population (OR = 2.018)^19^. Some studies have reported AG, GG or AG+GG genotypes are more susceptible to different cancers, particularly for esophageal/gastric cancer or esophageal squamous cell carcinoma compared to AA genotype ^20^^,^^21^ . In a meta-analysis study on 13676 patients with gastric cancer, it has been concluded that there is a significant relationship between *PLCE1 *rs2274223 polymorphisms and the incidence and increased risk of gastric cancer^   22 ^. Cui *et al*. investigated the relationship between four functional SNPs in *PLCE1 *gene (including rs12263737, rs2274223, rs11187842 and rs753724) and the risk of esophagus cancer in 222 Chinese cases and 326 controls^        23 ^. The results demonstrated that these polymorphisms are closely associated with higher risk of esophageal cancer. For these reasons, many researchers consider *PLCE1* gene polymorphisms as one of the significant biomarker involved in esophagus cancer among Chinese populations.

In other research, genetic variation in *PLCE1* and the risk of upper gastrointestinal cancers and esophageal adenocarcinoma was evaluated in Caucasian populations^        10 ^. The results showed that rs4072037 SNP in *PLCE1* is associated with higher risk of upper gastrointestinal cancer, while rs2274223 and rs4072037 were correlated with increased risk of esophageal adenocarcinoma. Similar results were also obtained in 108 cases with gastric cancer in Kashmir Valley population^   24 ^. A significant correlation was observed between three functional SNPs in *PLCE1* gene (rs2274223 A>G, rs3765524 C>T and rs7922612 C>T) and gastric cancer. The effect of three different SNPs in *PLCE1* gene (rs2274223A> G, rs3765524C>T and rs7922612C>T) and gastric cancer was also evaluated in 135 cases and 195 healthy controls in Kashmir Valley population^        14 ^. Although G2274223, T3765524, and T7922612 haplotypes were significantly correlated with higher risk of gastric cancer, these polymorphisms were not independently correlated with gastric cancer and several associated factors such as smoking and family history may be involved^        14 ^. In the present study, about 57% of cases were smokers and the family history of gastric cancer was 35%. Therefore, it can be suggested that these factors may also increase the risk of gastric cancer along with the suspected polymorphism genotypes. 

In a meta-analysis study, Zhang et al. analyzed the results of 13188 cases with gastric cancer and 14666 controls^   25 ^. They demonstrated that there is a relationship between rs2274223 A>G polymorphism (all genotypes of AA, AG and GG) and disease stage, which was most popular among Asian populations. In another investigation, the correlation between rs2274223 polymorphism was considered in 380 esophageal cancer patients and 380 healthy controls. The results revealed that the rs2274223 is significantly associated with increased risk of the cancer. Additionally, AG genotype in women was greater than that in men. For these reasons, some studies considered rs2274223 SNP as a sensitive biomarker for gastric cancer^   17 ^.

Although various studies found a significant correlation between SNPs in PLCE1 and the risk of cancers, there are several studies that did not find any significant correlation. For example, Ma et al. investigated the relation between rs2274223G, rs3203713G and rs11599672T polymorphisms in patients with head and neck cancers, but did not find any significant correlation^        26 ^.

## CONCLUSION

 In summary, in this case-control study, we investigated the correlation between the functional rs2274223 SNP in *PLCE1* gene and increased risk of gastric cancer for the first time in Iranian population. Although previous studies reported a close relationship between this SNP and enhanced risk of different cancers, we could not find a significant correlation between rs2274223 SNP in *PLCE1* gene and the risk of gastric cancer, tumor characteristics, and disease stage. Other factors such as smoking and family history may independently affect the risk of gastric cancer. However, additional larger studies among Iranian populations are needed to validate our findings. 
